# Evaluation of the Relationship Between Expression of Villin and Gelsolin Genes and Axillary Lymph Node Metastasis in Patients with Breast Cancer

**DOI:** 10.30699/ijp.2020.121532.2322

**Published:** 2020-10-10

**Authors:** Armin Borhan, Zohreh Nozarian, Alireza Abdollahi, Reza Shahsiah, Hadiseh Mohammadpour, Arash Borhan

**Affiliations:** 1 *Department of Pathology, Cancer Institute of Iran, Imam Khomeini Hospital, Tehran University of Medical Sciences, Tehran, Iran*; 2 *Iran National Tumor Bank, Cancer Institute of Iran, Tehran University of Medical Sciences, Tehran, Iran*

**Keywords:** Breast cancer, Gelsolin, Metastasis, Villin

## Abstract

**Background & Objective:**

Nowadays, actin-binding proteins such as Villin and Gelsolin have been considered to be associated with aggressive tumors. This study mainly aims to determine the relationship between Gelsolin and Villin genes expression and metastasis of axillary lymph nodes in patients with breast cancer.

**Methods:**

The included population consisted of 40 confirmed cases of female breast cancer (including 20 patients with breast cancer along with axillary lymph node metastasis and 20 patients without axillary lymph node metastasis). Expression of Villin and Gelsolin genes was evaluated using Real-time PCR and pre-designed primers.

**Results:**

The mean expression level of Villin in groups with and without axillary lymph node metastasis was 3.33±1.35 and 0.87±0.88, respectively (*P*<0.001). The mean Gelsolin expression levels in both groups (with and without axillary lymph node metastasis) were 4.13±2.40 and 1.00±0.35, respectively (*P*<0.001). The significant relationships were independent of individuals’ age.

**Conclusion:**

Patients with axillary lymph node metastasis may express significant higher level of Villin and Gelsolin genes.

## Introduction

Breast cancer is generally considered as the most common malignancy among the female population, affecting 1.7 million women worldwide every year (1, 2) constituting one-fourth of cancers in women and 15% of all cancer-related deaths (3). One in 8 women will develop breast cancer during life (4). In developing countries such as Iran, breast cancer rate increases by about 5% every year (5). Breast cancer onset happens10 years earlier in the Iranian female population (24 in 100000) (6, 7). Recently, the survival rate of breast cancer patients has significantly increased due to early detection of malignancy and multiple effective treatments including surgical approaches as well as various chemoradiotherapy agents (1, 8, 9). Villin, a 92.5 kDa protein, is a crucial member of actin-binding proteins generally found in the microvilli of brush borders of epithelial linings of different gastrointestinal organs such as stomach, pancreas gland, bile ducts and intestines; as well as corresponding adenocarcinomas (10, 11). It has been suggested that the expression of Villin gene is closely related to a higher rate of malignant tumors metastatic progression, aggressive behavior of cancer and consequently poorer prognosis (12). 

Gelsolin, a member of Gelsolin protein super family, is an 82 kDa protein likely expressed in various cell types containing six homologous subdomains, ultra-structurally. Gelsolin is found in intracellular and extracellular regions (13, 14). Variations in Gelsolin gene expression leads to cytoskeletal changes during cell differentiation and carcinogenesis (13, 15). Some of the previously conducted studies suggest the role of inhibiting Gelsolin gene in tumor suppression. Declined expression of the Gelsolin gene was related to invasive breast cancer in mentioned studies (16, 17).

As there are little studies regarding the role of Villin gene and Gelsolin gene in patients with breast cancer in the Iranian female population, we designed this study to evaluate the relationship between gene expression of Gelsolin and Villin and axillary lymph node metastasis in patients with breast cancer.

##  Materials and Methods

This cross-sectional descriptive-analytic study was conducted in Imam Khomeini hospital complex (affiliated hospital of Tehran University of medical sciences) between January 2017 and January 2018.

The inclusion criteria were: invasive ductal carcinoma of breast, grade 2 or 3. 

The exclusion criteria were: previous chemotherapy and/or radiotherapy. 

Informed consent forms were obtained from included cases.

A portion of each tissue sample was fixed in 10% formalin and subsequently blocked in paraffin. Hematoxylin and eosin stained slides were prepared for histopathological evaluation including tumor histology (type and grade) and presence or absence of axillary lymph node metastasis. It is necessary to mention that the tissue diagnosis of breast malignancy, as well as tumor histologic grade, was performed by histopathological evaluation, utilizing Bloom-Richardson grading system (18).

The other portion of tissue sample was freshly received and stored in liquid nitrogen for RNA extraction. To determine the level of mRNA of each of the Villin and Gelsolin indices, the total RNA from the mentioned frozen tissue sample was extracted using the Invitrogen TRIzol kit (Thermo Fisher Scientific, United States), as stated by the following procedure. Tissue samples were homogenized and vortex mixed for 10 seconds followed by addition of 200 μL of Chloroform. Samples were centrifuged for 15 minutes at 1200 rpm and 4°C. The supernatant component was gathered and centrifuged after adding 500 μL of isopropanol. The RNA pellets were washed using Ethanol and air dried. Subsequently, the RNA pellets were eluted with Diethyl Pyrocarbonate (DEPC) treated water. Afterwards, extracted RNA was quantified using a NanoDrop ND-2000 spectrophotometer (NanoDrop Technologies, Wilmington, DE). Quality validation of the extracted RNA was also done by gel electrophoresis technique. Using the RevertAid First Strand cDNA synthesis kit (Thermo Fisher Scientific, United States), complementary DNA (cDNA) was generated from mRNA. Mentioned synthesized cDNA was evaluated with housekeeping gene control. To monitor gene expression of two indices of the study (Villin gene and Gelsolin gene), real-time PCR (Takara Bio Inc., Japan) was performed using pre-designed primers (Table 1). Finally, values were determined from two indices in two groups of study.

**Table 1:** Pre-designed primer sequences used to determine genes expression (19)

Gene name	Primer sequence
**Villin**	F: 5'-GGCCAGCCAAGATGAAATTA-3' R: 5'-CTCAAAGGCCTTGGTGTTGT-3'
**Gelsolin**	F: 5'-AGGTGGAGGAAGGCAGTGAAC-3' R: 5'-CGAAGAGGCGAGGAGGATGAG-3'

We applied SPSS software (version 19.0, IBM Corp., Armonk, NY, USA) for statistical data analysis. Mann-Whitney U test was also applied to contrast continuous variables. Statistical significance was set at P-value<0.05.

## Results

**Fig. 1:**
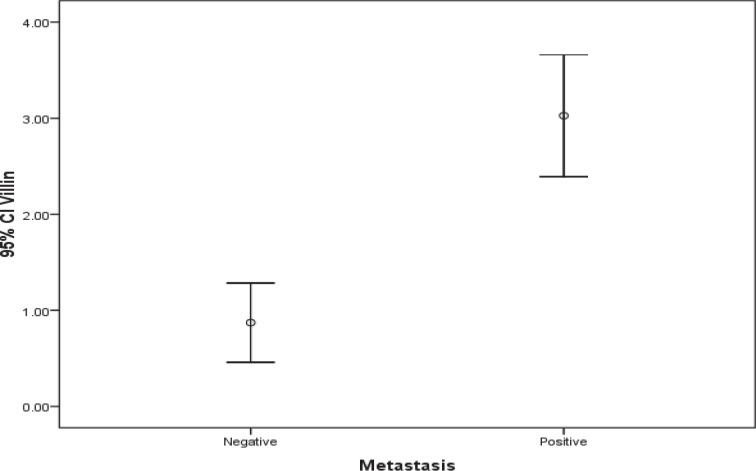
Comparison of Villin gene expression in both groups

**Fig. 2:**
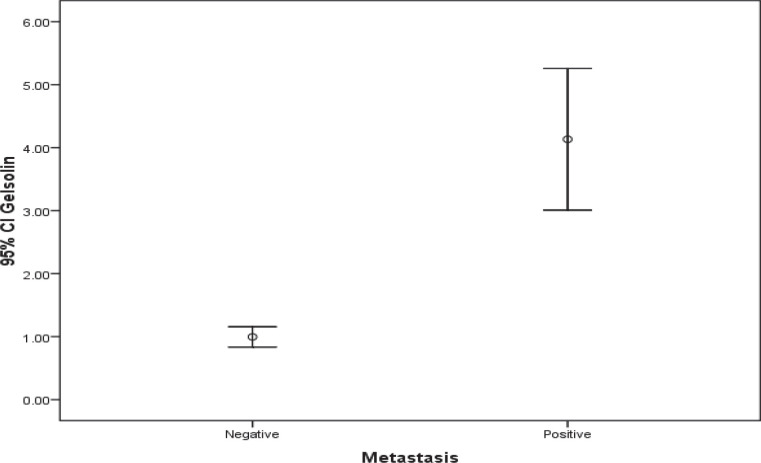
Comparison of Gelsolin gene expression in both groups

The population involved in this study included 40 women with histologically confirmed breast cancer and mean age of 59.4±10.6 (36-78) years. Half of the patients were also dealing with axillary lymph node involvement. There was a significant difference regarding Villin and Gelsolin gene expressions in patients with and without axillary lymph metastasis (Table 2) (Figures 1 and 2).

We did not detect a significant correlation between Villin and Gelsolin gene expression levels and patients’ age in our study (Table 3).

**Table 2:** Comparison of Villin and Gelsolin genes expression in both groups

	With metastasis	Without metastasis	P-value
Villin	3.03±1.35	0.87±0.88	<0.001
Gelsolin	4.13±2.4	1±0.35	<0.001

**Table 3:** Correlation between Villin and Gelsolin levels and patients’ age

	With metastasis	Without metastasis
Villin	R=0.15, *P*=0.51	R=0.12, *P*=0.59
Gelsolin	R=0.021, *p*=0.9	R=0.93, *P*=0.18

## Discussion

The results of this study were indicative of significantly higher levels of Villin and Gelsolin genes expressions in breast cancer patients with axillary lymph node metastasis rather than cases without axillary lymph node metastasis. We also found that their expression level was not significantly correlated with patients’ age thus the expression levels of the mentioned genes were higher in both old and young affected cases.

In a previous study, Baig *et al.* analyzed the expressional profile of Gelsolin gene in tissues obtained from human breasts. They found that the expression level of the Gelsolin gene was down-regulated in breast cancer tissues and was associated with metastatic growth as well as death in breast cancer cases. In other words, Gelsolin gene expression was lower in the breast cancer group compared to control group and mismatching our findings, the level was even lower in patients with metastatic involvements (8). 

In another study, Tutunchi *et al.* assessed Gelsolin gene expression in 70 female breast cancer cases suggesting an increase in Gelsolin gene expression in breast cancer patients. Overexpression of Gelsolin gene is a leading cause of axillary lymph node metastasis in patients with breast cancer. There was not a significant relationship between Gelsolin gene expression levels and tumor size or tumor histologic grades (20).

Another study by Zhu *et al.* investigated the expression levels of five types of peptides including Gelsolin in patients with non-small cell lung cancer (NSCLC). They noticed that the increased Gelsolin gene expression level was associated with lymph node metastasis, malignant disease progression as well as increased risk of mortality (21).

Abedini *et al.* assessed the relationship between Gelsolin gene expression and clinical prognosis as well as survival in women with ovarian serous adenocarcinoma. They reported higher Gelsolin gene expression in women with ovarian cancer which was associated with more tumor growth and poorer clinical prognosis. In other words, they realized that Gelsolin overexpression was notably associated with more invasive tumor behavior and higher cancer mortality rates (22).

The results also suggested significantly higher levels of Villin gene expression in patients with lymph node metastasis than the other group.

In a study by Mohammadpour *et al.*, 42 breast cancer patients’ samples were investigated and expression levels of Villin and HER-2 genes were detected with quantitative PCR using pre-designed primers. They realized that Villin gene expression was positivity correlated with HER-2 and aggressive tumor nature. Hence, Villin gene overexpression may be a superior predicting element in invasive breast cancer cases (19).

Ma *et al.* studied the relationship between Ezrin protein (Villin) expression and the carcinogenesis as well as prognosis of breast invasive ductal carcinoma. They found marked Ezrin protein (Villin) expression in breast invasive ductal carcinoma which was positively associated with axillary lymph node involvement. They also suggested that high expression levels of Ezrin protein (Villin) may be utilized as a good predicting factor for unfavorable prognosis in the patients with infiltrating ductal breast carcinoma (23).

Yang also conducted a study evaluating Villin gene expression in 52 breast ductal carcinoma patients. Contrary to our results, he found that Villin was absent in all breast cancer cases (24). 

Gelsolin and Villin have basic roles in cytoskeleton regulation. Gelsolin is associated with motility, apoptosis, and is indicative of cancer phenotype while it is considered to be an activator and an inhibitor of apoptosis in different cancers (13, 22, 25).

Our results reveal that Gelsolin gene expression is significantly higher in breast cancer cases with axillary lymph node metastasis which highlights greater stages of the disease in these cases. In addition, elevated Gelsolin gene expression level is not correlated with patients’ age.

Villin has a crucial role in actin reorganization and cell remodeling so that it affects cancerous cells migration and invasion (26, 27). Villin is an actin-binding protein expressed in several organs of gastrointestinal system such as stomach, pancreas gland, bile ducts and intestines and its higher concentrations are also detected in kidney, lung, endometrium, and ovary adenocarcinomas (28-30).

Our findings highlight that Villin gene expression is significantly higher in axillary lymph node metastatic breast cancer patients in comparison with those without lymphatic involvement, and it is not correlated with patients’ age either.

This study had some limitations. Firstly, it was conducted in a tertiary center. Secondly, our sample size was limited. Thirdly, we did not evaluate hormone receptors, HER2 as well as other genes expressions in our study. So larger multi-centric studies along with evaluating the expression of the more genes are recommended. In other words, future investigations will be needed to reach more comprehensive correlations and results.

## Conclusion

Villin and Gelsolin genes expression were significantly higher in female breast cancer patients with axillary lymph node metastasis in comparison with breast cancer patients who did not deal with such involvement.
